# Comment on Re-Analysis of Data by Terluin, de Boer and de Vet

**DOI:** 10.1371/journal.pone.0162329

**Published:** 2016-11-23

**Authors:** Johanna Wigman, Jim van Os, Marieke Wichers

**Affiliations:** 1 University Center for Psychiatry, University of Groningen, University Medical Center Groningen, Groningen, The Netherlands; 2 Mental Health Service (GGZ) Friesland, Leeuwarden, The Netherlands; 3 Department of Psychiatry and Psychology, Maastricht University Medical Centre, Maastricht, The Netherlands; 4 King’s College London, King’s Health Partners, Department of Psychosis Studies, Institute of Psychiatry, London, United Kingdom; Katholieke Universiteit Leuven, BELGIUM

The disputing paper by Terluin, de Boer and de Vet [1] that was based on re-analysis of our data presents conclusions that contrast with the findings in our original paper. Their paper raises several important points that are relevant not only for our study but for the field at large. The problem they raise is that of unequal variances in the subgroups due to skewness of the data. This point is indeed important and was not addressed appropriately in our original paper. As Terluin and colleagues reported, the variances of the ESM variables increased with increasing severity groups, which we had not inspected in the original work. We agree that, given these new insights, it cannot be ruled out that differences in variance may account for between-group differences in connection strength.

Based on Terluin et al.’s results, it can thus be stated that our conclusions have “not been unequivocally confirmed”. However, it should be kept in mind that (i) although they tried to replicate our analyses as closely as possible, they took several *ad hoc* steps during their analysis process (e.g. grand mean centering; rescaling the scores) making it difficult to directly compare their results to our original results and to results that might have been obtained with other sets of preprocessing steps/analytic choices; and (ii) they focused only on our conclusions regarding staging but not regarding profiling. Based on this, we argue that our conclusions are not unequivocally disconfirmed either. Moreover, the ideas put forward in our original paper have been valuable as first steps into the field of longitudinal mental-state network modelling.

We agree with the authors on the importance of using appropriate methods in psychological research and acknowledge that, in retrospect, some of the analytic choices we made at the time of the original work were not optimal. However, it should be noted that the original article, published in 2013 [2], was pioneering work regarding the empirical testing of longitudinal dynamic networks in psychopathology. At the moment of publication, the use of multilevel time-lagged analyses in the context of network theory was quite still novel and analytic techniques and strategies were still being developed and optimized. Research is ever a process of working to the best of one’s knowledge, only to find out later that better, more optimal or improved methods exist.

Most psychopathology research in general population samples faces the problems of non-normally distributed measures and unequal variances across subgroups. The data in our original study form an illustrative example of these issues but are, however, by no means unique for the field. As such, we agree with the authors that the issue of unequal variance needs proper attention in all future psychopathology research.
